# Protective effect of crocin on electromagnetic field-induced testicular damage and heat shock protein A2 expression in male BALB/c mice

**DOI:** 10.22038/IJBMS.2019.38896.9229

**Published:** 2020-01

**Authors:** Shayan Vafaei, Fatemeh Motejaded, Alireza Ebrahimzadeh-bideskan

**Affiliations:** 1 Department of Anatomy and Cell Biology, School of Medicine, Mashhad University of Medical Sciences, Mashhad, Iran; 2 Microanatomy Research Center, Mashhad University of Medical Sciences, Mashhad, Iran

**Keywords:** Apoptosis, Crocin, Electromagnetic field, Heat shock protein, Testis

## Abstract

**Objective(s):**

Exposure to electromagnetic fields (EMF) emitted from mobile phones may cause a deleterious effect on human health and may affect the male reproductive system. *Crocin*, a carotenoid isolated from *Crocus Sativus L. (Saffron)*, is a phar¬macologically active component of saffron. So, this study was conducted to investigate the protective effect of *crocin* on the male reproductive system of 60 day old mice after EMF exposure.

**Materials and Methods:**

Twenty-four male BALB/c mice were randomly divided into 4 groups: 1. Em group (2100 MHZ); 2. Cr group (50 mg/kg); 3. Em+Cr group (2100 MHZ+50 mg/kg), and 4. Control group. Sperm parameters (count, and abnormal percent), testis weight index, testis volume, seminiferous tubule diam¬eter, germinal epithelium thickness, LH, FSH and testosterone serum level, testicular Heat shock protein A2 (HspA2) immunoreactivity, and apoptosis were evaluated.

**Results:**

HspA2 immunoreactivity, apoptosis in the germinal epithelium and abnormal sperm were increased in Em group compared with the control group (*P*<0.05). Sperm count, LH, and testosterone serum level were decreased in the Em group compared with the control group (*P*<0.05). These parameters were improved in the Em+Cr group compared with Em group significantly (*P*<0.05).

**Conclusion:**

our findings revealed that EMF exposure leads to harmful impressions on the male reproductive system, while crocin can attenuate EMF-induced destructive effects.

## Introduction

Nowadays with the progress of communication technology and development of communicating devices, the use of equipment for connecting people to each other is increasing. As a result, exposure to electromagnetic fields (EMF) is growing. Accumulating evidence has shown that EMF potentially induces negative effects on human health (1). Mobile phones send and receive signals via EMFs that are absorbed by its user (2). Most 2G phones use the Global System for Mobile Communications (GSM) standard, which pulses 900–1800 MHz signals, whereas most newer phones use the Universal Mobile Telecommunications System’s (UMTS), which operates at a higher frequency range of 1900–2170 MHz and is without periodic pulsed modulation content. There is evidence that the frequency components may be very significant for mobile phone-related biological effects (3). It has been proven that the wave frequency and duration of exposure are the main factors which influence the harmful effects EMF on the male reproductive system (4, 5). Previous studies have concluded that EMFs have probable harmful effects on Leydig cells, seminiferous tubules, and spermatozoa. In addition, exposure to EMF may cause testosterone reduction biosynthesis, sperm DNA damage, and impair spermatogenesis (6, 7). Other studies showed that cell phone radiation may cause structural and functional injuries of the testis, alter semen parameters, and reduce epididymal sperm concentrations (8).

Previous studies demonstrated that exposure to EMF will disrupt the function of heat shock proteins (Hsp) (9). Expression of Hsp is one of the cellular reactions to stress (10). These proteins are released into the extracellular space in stressful conditions. Also, these macromolecules, by maintaining protein homostasis, can prevent apoptosis(11). Any fault in protein expression, especially in the late phase of the spermiogenesis, leads to DNA damage. In this regard, HspA2 can be considered a biochemical marker for sperm function and male fertility (12, 13).

A large, increasing number of patients worldwide use medicinal plants and herbs for health purposes (14). Crocin is a carotenoid obtained commercially from the dried trifid stigma of *Crocus sativus *L*. (Saffron*). This agent is responsible for the red color of saffron (15, 16). Throughout history, traditional medicine recommended saffron to treat infertility, impotence, and as a sexual potential stimulant (17). In addition, recent studies have proven that crocin has pharmacological and therapeutic activities such as antidepressant (18), anti-inflammatory (19), anticarcinogenic (20), anti-oxidant (21), radical scavenging neuron-protective, geno-protective (22), and memory-improver (23). For example Salahshoor *et al.* in 2016 noticed that crocin administration can reduce the adverse effects of nicotine on serum testosterone level and reproductive parameters (sperm count, viability, motility, testis weight, and seminiferous tubule diameters) in male mice (24).

In this regard, we decided to study the adverse effects of exposure to EMF and the therapeutic effect of crocin*. *So in this study, the effect of 2 hr/day EMF exposure and the therapeutic effect of crocin on the spermatogenic cell line, serum biochemical parameters, and some semen features were studied.

## Materials and Methods

This study was approved by the Ethical Committee (IR.MUMS.MEDICAL.REC.1397.488) for the Care and Use of Laboratory Animals at Mashhad University of Medical Sciences, Mashhad, Iran.

***Animals***

For this study, 24 male Bulb/c mice (60 days old and weighing 30–40 g) were obtained from the Animal Center of the faculty of medicine, Mashhad University of Medical Science. The animals were maintained under standard laboratory conditions in an air-conditioned room, where the temperature was maintained at 25 °C with constant humidity (40–50%) and kept on a 12/12 hr light/dark cycle and free access to food and water throughout the experiment (25).

***Crocin*** ***preparation***

Crocin powder was purchased from the Faculty of Pharmacy, Mashhad University of Medical Sciences and kept in a dry and dark place. For intraperitoneal injection, in each day, crocin was dissolved in distilled water at 10 mg/ml concentration. The volume of injection was adjusted according to the varying weights of mice (50 mg/kg) (24, 26).

***Electromagnetic field (EMF)***

**Figure 1:**
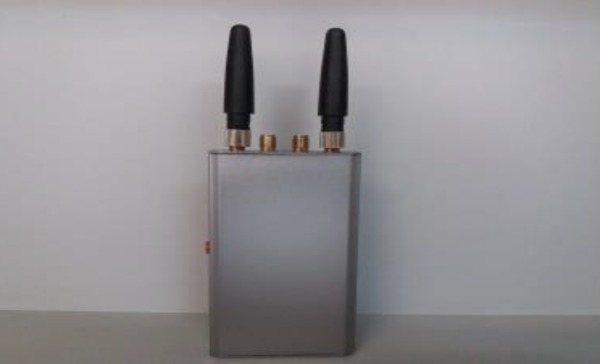
Electromagnetic field-producing device (2100 MHZ)

In this study, the electromagnetic field was created with a device (Figure 1) that produced a 2100 MHZ electromagnetic field like 4G-LTE mobile phones. 

***Experimental design***

Twenty-four mice were randomly divided into 4 groups:

1) Em group; the animals were exposed to 2100 MHZ of EMF for 2 hr in each day and injected distilled water (1 ml/100 g of body weight) for 16 consecutive days (27). 

2) Em+Cr group; the animals were exposed to 2100 MHZ of EMF for 2 hr in each day and injected crocin (50 mg/ kg/ daily) for 16 consecutive days (24, 26).

3) Cr group; the animals were injected crocin (50 mg/ kg/ daily) for 16 consecutive days (24, 26). 

4) Control group; without any intervention. 

At the end of the experiments, the animals were weighed; blood samples were collected and then sacrificed to remove their testes and epididymis. The left testes were weighted using scales and then fixed in 10% neutral buffered formalin (NBF) for 4 days.

***Weight index and volume of the testis***

The following formula was used to calculate the weight index of the testis:

WI=(Testis Weight g)(Animal body weight g)×100

Testis volume was calculated using the following formula:

$\mathrm{Vt}=\frac{\left(\mathrm{W2}-\mathrm{W1}\right)}{\delta }$

W1= Initial weight (beaker + normal saline + basket)

W2= Secondary weight (beaker + normal saline + basket + testis)

δ = Density of normal saline (1.0048) (28, 29).

***Sperm count and morphology***

The epididymis was cut into three pieces and placed in 2.5 ml of normal saline and incubated at 37 °C for 15 min. After that 10 µl of the solution was placed on a Neubauer slide and sperm in four squares was counted under a light microscope (Olympus BX51) with the 40×objective lens and mean of the counted sperm was reported. The number of sperm in 1 ml was calculated using the following formula:

$n=\frac{(\mathrm{mean of sperms in four square}\times 2.5}{\mathrm{volume of one square}(100\mathrm{nL})}$

For abnormal sperm assessment, the smear was prepared (50 µl of the epididymal sperm solution was placed on a slide and fixed with methanol 70%) and Papanicolaou staining was done. Sperm morphology was evaluated on each slide and abnormal sperm (sperm without tails, sperm with coiled or bent tails, and sperm with dual and abnormal head) were counted using an optical microscope (28, 30).

***Biochemical analyses***

The collected blood samples were centrifuged and serum was separated. The concentration of testosterone, luteinizing hormone (LH), and follicle stimulating hormone (FSH) were measured using the enzyme-linked immunosorbent assay (ELISA) method. We used commercially available human ELISA kits (My Biosource, USA). The procedure for the ELISA method was followed according to the instructions provided by the manufacturer. The serum levels of testosterone, LH, and FSH were presented as ng/dl (31).

***TUNEL method***

Terminal deoxynucleotidyl transferase dUTP nick end labeling (TUNEL) method was used to detect apoptotic cells by means of a TUNEL Kit (Roche 11684817910). To do TUNEL assay, NBF-fixed, paraffin-embedded tissue specimens were cut into 5 µm thicknesses. After deparaffinization and dehydration, in order to neutralize the endogenous peroxidase, samples were placed in 3% hydrogen peroxide solution in ethanol for 15 min. After washing, the tissue sections were incubated with protein kinase K for 15 min. Next, the tissue sections were washed three times with Phosphate Buffer Saline (PBS) and incubated with the reaction solution of the TUNEL staining kit at 4 ^°^C overnight. After washing the samples were incubated with 3,3’-Diaminobenzidine (DAB) solution for 15 min at room temperature and were then stained with hematoxylin. In this method, the cells with brown nucleus were evaluated as TUNEL-positive cells.

Different regions of the prepared testicular tissue sections were photographed using a microscope (Olympus BX51, Japan), 40x objective lens, and equipped with a high-resolution camera, and the images were transferred to a computer. The numbers of apoptotic cells were counted by using rectangular grids placed randomly in the investigated areas. Cells with the nucleus in dark brown color were considered apoptotic cells. Stereological methods were applied to count apoptotic cells. Finally, the mean numbers of apoptotic cells per unit area (NA) were calculated using the following formula (32):

Where, ‘NA’: number of TUNEL positive cells per unit area, ‘ΣQ’: the total counted TUNEL positive cells, ‘a/f’: area of every frame counted, and ‘ΣP’: the total number of frames that have been in collision with section (28, 33, 34).

***Immunohistochemistry***

This method was performed for localization of HspA2. For this technique, NBF-fixed, paraffin-embedded, 5 µm thick sections were deparaffinized by xylene, rehydrated by descending grades of ethanol, and washed with 0.1 M PBS for 15 min. Then, antigen retrieval was performed at room temperature for 20 min by 0.01% protein kinase (10 µl of protein kinase in 1 ml of 0.1 M PBS), followed by washing with 0.1 M PBS for 15 min. Afterward, 1% bovine serum albumin (BSA) solution was prepared in PBS (1 g BSA in 100 ml of 0.1 M PBS), and 100 ml Triton X-100 was added in order to place 30–50 ml of the obtained combination on each section. In the next stage, 3% methanol/H2O2 solution was used to block endogenous peroxidase, so that the samples were placed in the solution in a dark, closed chamber for 10–15 min. Tissue sections were incubated with primary antibodies (Abcam Co., Cambridge, MA; Cas. No. 154374) in a humid chamber at 4 °C overnight. After washing in PBS, the tissue sections were incubated with HRP conjugated secondary antibody (Abcam Co., Cambridge, MA; Cas. No. 97051) of 37 °C for 1.5 hr at room temperature in a closed humid chamber. Following that, 0.03% DAB (0.03 g in 100 ml of PBS and 200 µl of H2O2/100 ml PBS) was added to the samples (10 min at room temperature). After rinsing with running water, Hematoxylin staining (15–20 sec) was performed, and after dehydration with ascending ethanol degrees and clearing with xylene, samples were prepared for microscopic examinations. Immunostained sections were evaluated and photographed using a light microscope (Olympus BX51, Japan). Twenty photos were taken from each testis. Based on staining intensity, sections were graded by using Likert spectrum (weak reaction = +‏, moderate reaction = ++, severe reaction = +++, ‏‏‏ and very severe reaction = ++++) (35-38).

***Measurement of seminiferous tubule diameter and germinal epithelium thickness***

To assess the diameter of the seminiferous tubules, ten images were randomly taken from each slide, using a microscope (Olympus BX51, Japan), 40x objective lens, and equipped with a high-resolution camera (dp12), and the images were transferred to a computer. Then, using the Analysis Image Processing Software (Olympus Co. LTD, Tokyo, Japan) seminiferous tubule diameter and germinal epithelium thickness were measured. 

***Statistical analysis***

The data were analyzed by one-way ANOVA and the Tukey’s *post hoc* tests and non-parametric Kruskal–Wallis and Mann–Whitney *U*-tests by means of SPSS software version 16. Values are expressed as means ± standard error of the mean (SEM) and *P*<0.05 was considered statistically significant. 

## Results

***Weight index of the testis***

**Figure 2:**
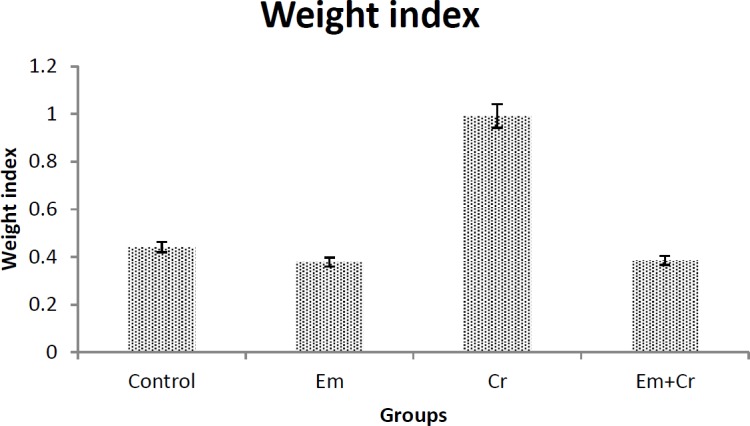
Comparison of the means of testis weight index in different groups showed that there were no significant differences between the studied groups

Comparison of the means of testis weight index in all groups showed that the testis weight index was decreased in the EM group compared with the control group and also testis weight index increased in the Cr group compared with the control group, but these changes were not significant (Figure 2).

***Testis volume***

**Figure 3:**
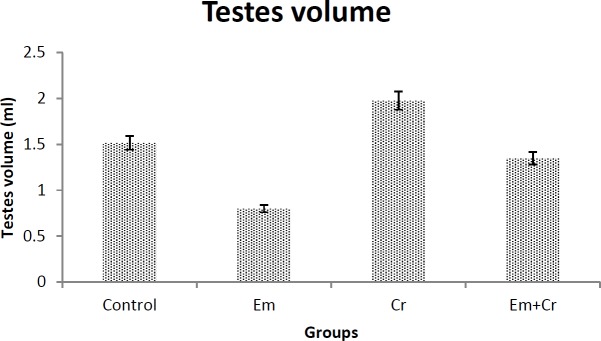
Comparison of the testis volume in different groups showed that there was no significant difference between the studied groups

The volume of testis was measured just after animal sacrifice. In comparison to the control group, testis volume was decreased in the EM group, while it increased in the Cr compared with the control and EM+Cr groups, but these changes were not significant (Figure 3).

***Sperm parameters***

**Figure 4:**
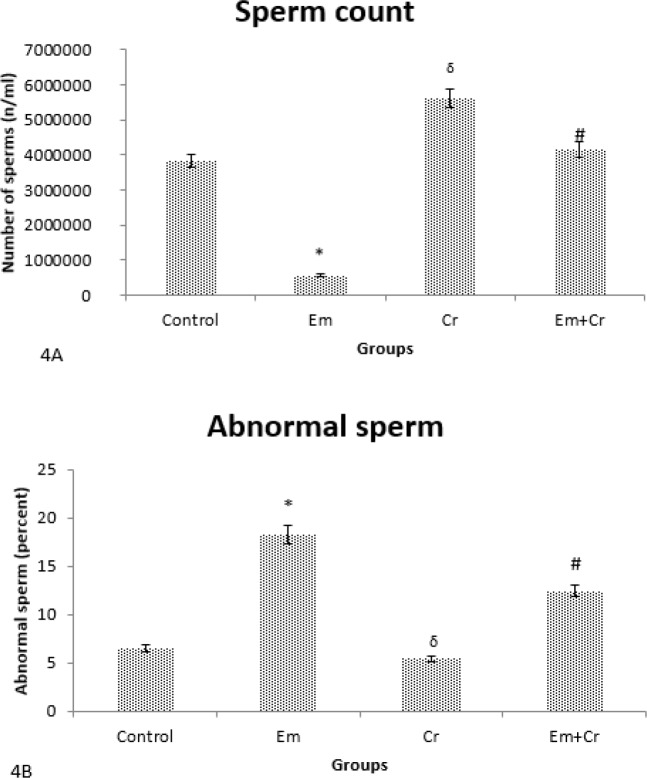
A. Comparison of the means of sperm counted in different groups showed that number of sperm in the Em group was decreased significantly compared with the Control group (*P<0.05) and increased in Em+Cr (#*P*<0.05) and Cr groups (δ*P*<0.05) compared with the Em group significantly

There was a significant decrease in the epididymal sperms in the Em group compared with the control group (*P*<0.05). Also, the number of epididymal sperms showed a significant increase in Em+Cr and Cr groups compared with the Em group (*P*<0.05), (Figure 4A).

Assessment of sperm abnormalities showed that the percentage of abnormal sperm was increased significantly in the Em group compared with the control group (*P*<0.001) but percentage of abnormal sperm of Em+Cr and Cr groups were increased compared with the Em group (*P*<0.05). There was a reduction in abnormal sperm percentage in the Cr group compared with the control group but this difference was not significant (Figure 4B).

***Biochemistry analyze***

**Figure 5:**
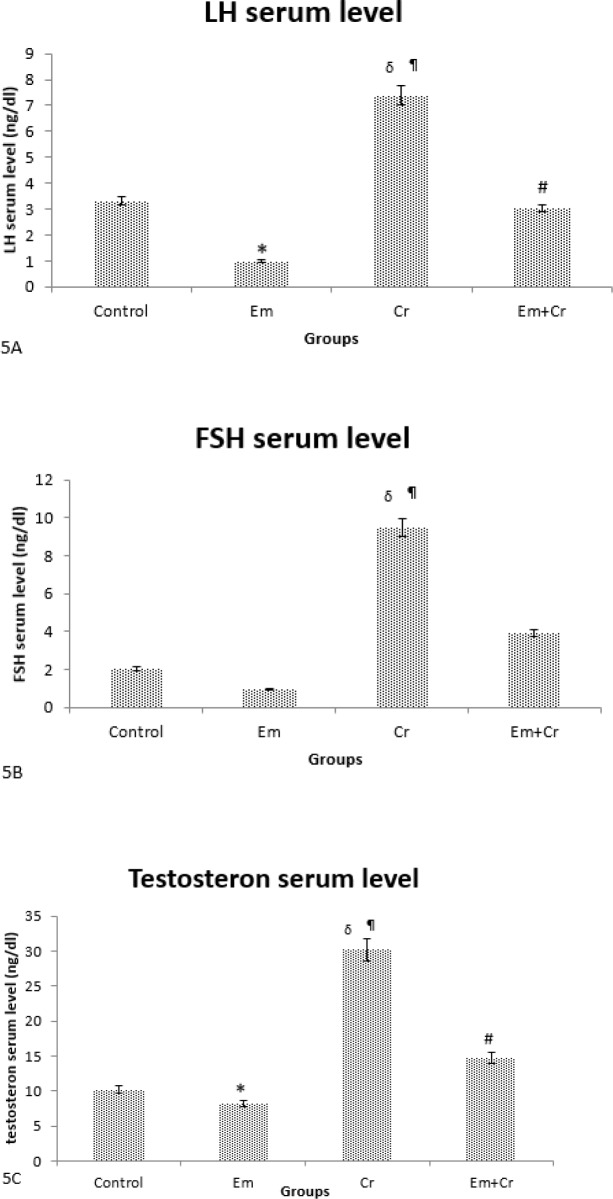
A. The mean of serum LH level was decreased significantly in the Em group compared with the control group (*P<0.001) and increased significantly in Em+Cr and Cr groups compared with the Em group (#*P*<0.001, δP<0.001, respectively), while in the Cr group it increased significantly compared with the Control group (¶*P*<0.001). B. The mean of serum FSH level in the Cr group increased significantly compared with Control and Em groups (¶*P*<0.001, δ*P*<0.001, respectively). C. Comparison of the mean of serum testosterone level in different groups showed that testosterone decreased significantly in the Em group compared with the control group (*P<0.05) and increased significantly in Em+Cr and Cr groups compared with the Em group (#*P*<0.001, δ*P*<0.001, respectively), while in the Cr group testosterone increased significantly compared with the Control group (¶*P*<0.001)

Serum LH level was decreased in the Em group compared with control group (*P*<0.05) and increased significantly in the Cr group compared with the control group (*P*<0.001), while it increased in Em+Cr and Cr groups compared with the Em group (*P*<0.001), (Figure 5A).

Serum FSH level was decreased in the Em group compared with the control group, but this reduction was not significant. Serum FSH level was increased significantly in the Cr group compared with control and Em groups (*P*<0.001). Although serum FSH level was increased in the Em+Cr group compared with the Em group, this increase was not significant (Figure 5B).

Serum testosterone level was decreased significantly in the Em group compared with the control group (*P*<0.05). Also, the serum testosterone level was increased significantly in the Cr group when compared with the control group (*P*<0.001), while serum testosterone level was increased in Em+Cr and Cr groups compared with the Em group (*P*<0.001), (Figure 5C).

***Histological measurements***

**Figure 6:**
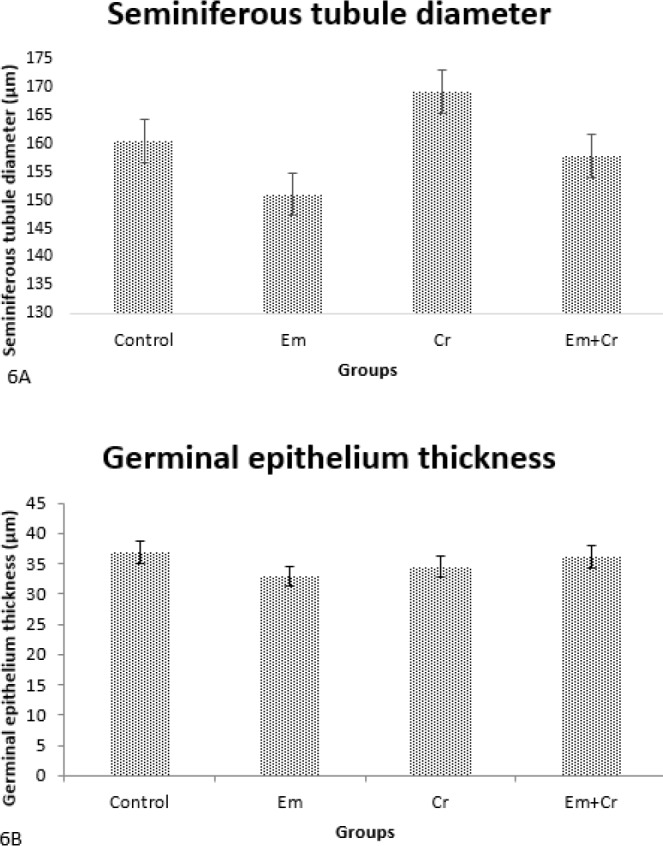
A. Comparison of seminiferous tubule diameters in different groups showed that the changes in the seminiferous tubule diameters between the studied groups were not significant. B. Comparison of the means of germinal epithelium thickness in different groups showed that the changes in the germinal epithelium thickness between the studied groups were not significant

The diameters of seminiferous tubules were decreased in the Em group compared with the control group and increased in the Em+Cr group compared with the Em group, but these changes were not significant (Figure 6A). Also, germinal epithelium thickness was decreased in the Em group compared with the control group and increased in the Em+Cr group compared with the Em group, but these changes were not significant (Figure 6B).

***TUNEL method***

**Figure 7:**
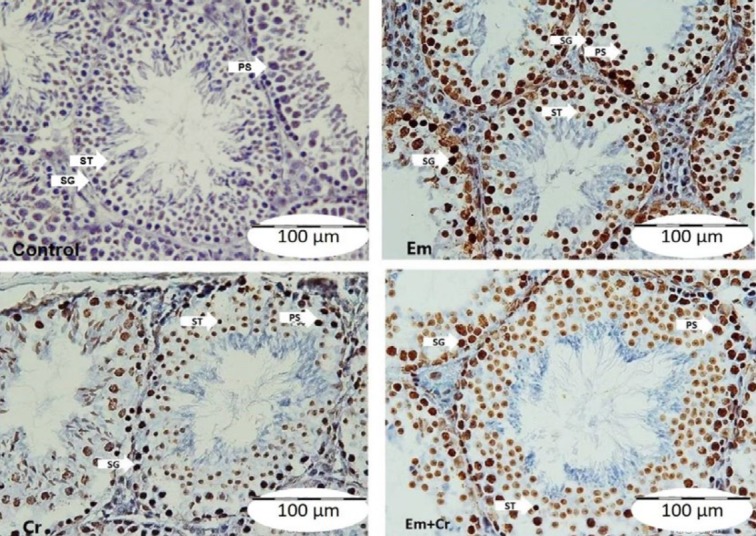
Photomicrographs of testicular tissue sections show the TUNEL positive cells (dark nucleus) in the germinal epithelium of studied groups. A; control group, B; Em group, C; Cr group, D; Em+Cr group. SG: spermatogonia, PS: primary spermatocytes, ST: spermatids, scale bar= 100 µm. Cr: Crocin; Em: electromagnet field

Our finding showed that the TUNEL positive cells (dark brown nucleus) were visible in testicular tissue sections of the Em group more than other groups (Figure 7).

**Figure 8:**
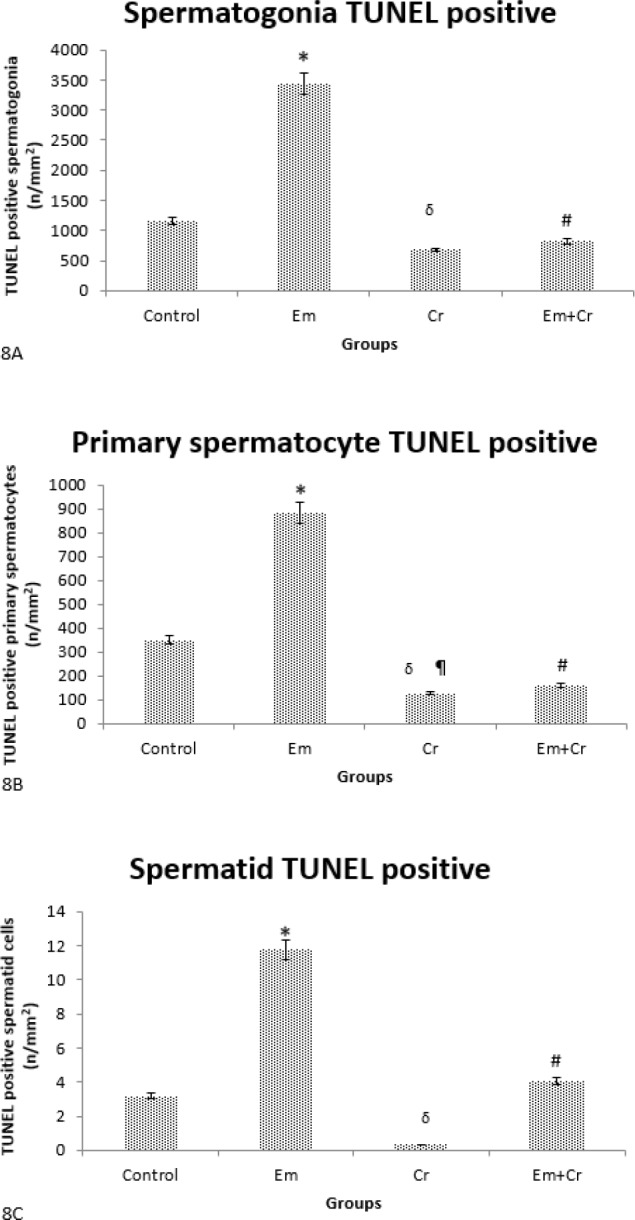
A. Comparison of the mean number of TUNEL positive spermatogonia in different studied groups. TUNEL positive spermatogonia increased significantly in the Em group compared with the control group (*P<0.001). Also, the number of TUNEL positive spermatogonia decreased significantly in Em+Cr and Cr groups compared with the Em group (#*P*<0.001, δ*P*<0.001, respectively). B. Comparison of the mean number of TUNEL positive primary spermatocytes showed that these cells increased significantly in the Em group compared with the control group (*P<0.001). Also, the number of TUNEL positive spermatocytes decreased significantly in Em+Cr and Cr groups compared with the Em group (#*P*<0.001, δ*P*<0.001, respectively) and decreased significantly in the Cr group compared with the control group (¶*P*<0.001). C. Comparing the mean number of TUNEL positive spermatids in the germinal epithelium of different studied groups showed that these cells increased significantly in the Em group compared with the control group (*P<0.001) and decreased significantly in Em+Cr and Cr groups compared with the Em group (#*P*<0.001) and (δ*P*<0.001). Cr: Crocin; Em: electromagnet field

The number of TUNEL positive spermatogonia was increased significantly in the Em group compared with the control group (*P*<0.001) while there was a significant reduction in these cells in Em+Cr and Cr groups compared with the Em group (*P*<0.001), (Figures 7, 8A). Also, the TUNEL positive primary spermatocytes were increased significantly in the Em group compared with the control group (*P*<0.001) and decreased significantly in Em+Cr and Cr groups compared with the Em group (*P*<0.001), (Figures 7, 8B). The numbers of TUNEL positive spermatid were significantly increased in the Em group compared with the control group (*P*<0.001), while there was a significant reduction in the number of these cells in Em+Cr and Cr groups compared with the Em group (*P*<0.001), (Figure 8C).

***Immunohistochemistry***

Heat shock protein (HspA2) immunoreactivity was evaluated in order to determine the expression of HspA2 in testis germinal epithelium of the different studied groups.

**Figure 9:**
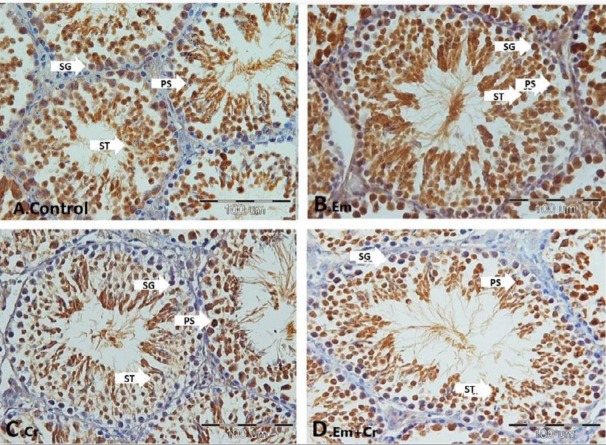
Photomicrograph of testicular germinal epithelium sections incubated with anti- HspA2 in different studied groups: A: control group, B: Em group, C: Cr group, D: Em+Cr group. SG: spermatogonia, PS: primary spermatocytes, ST: spermatids, scale bar= 100 µm

**Figure 10:**
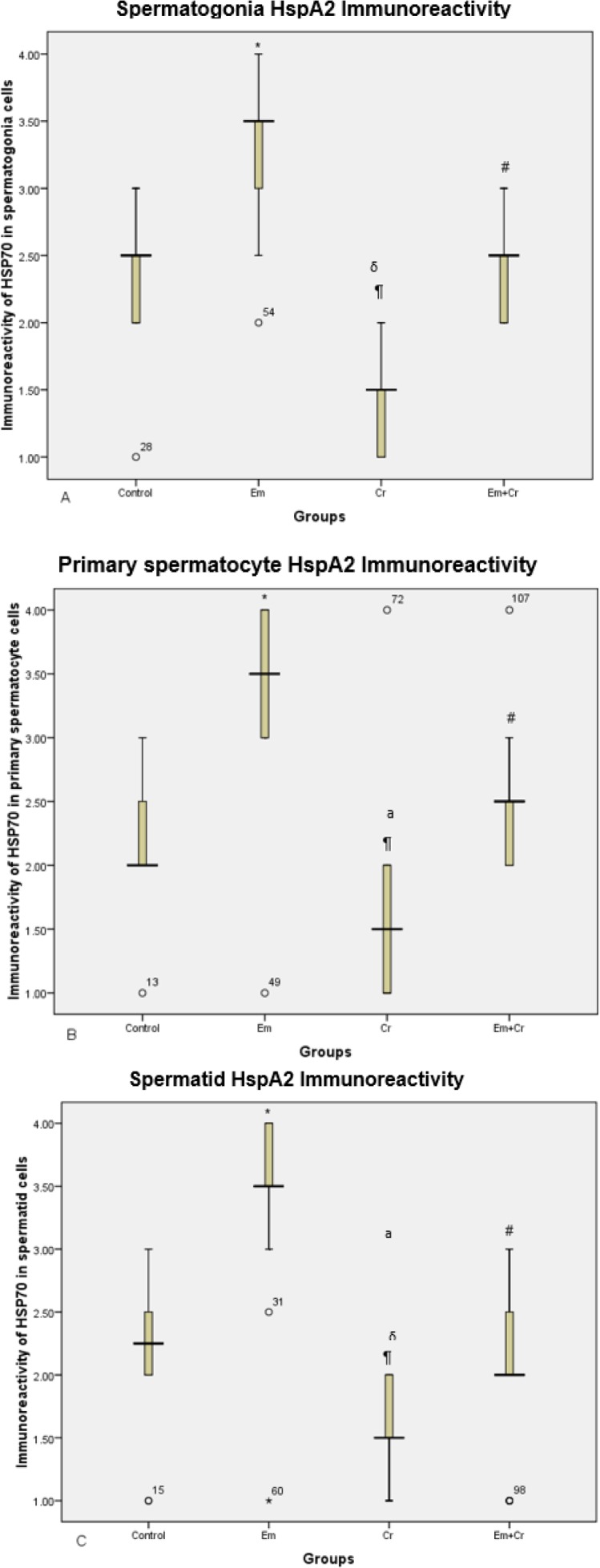
A. Comparison of HspA2 immunoreactivity in spermatogonia showed a significant increase in the Em group compared with the control group (*P<0.001) and decrease in Em+Cr and Cr groups compared with the Em group (#*P*<0.001) & (δ*P*<0.001), while this reaction decreased significantly in the Cr group compared with the control group (¶*P*<0.001). B. Comparison of HspA2 immunoreactivity in primary spermatocytes showed a significant increase in the Em group compared with the control group (*P<0.001) and decrease in Em+Cr and Cr groups compared with the Em group (#*P*<0.001) & (δ*P*<0.001), while this reaction decreased significantly in the Cr group compared with the control group (¶*P*<0.001). C. Comparison of HspA2 immunoreactivity in spermatid showed a significant increase in the Em group compared with the control group (*P<0.001) and significantly decreased in Em+Cr and Cr groups compared with the Em group (#*P*<0.001) & (δ*P*<0.001), while immunoreactivity of HspA2 in spermatid decreased significantly in the Cr group compared with the control group (¶*P*<0.001)

Our results showed that HspA2 immunoreactivity was increased significantly in different cells of germinal epithelium including spermatogonia (*P*<0.001), primary spermatocytes (*P*<0.001), and spermatids (*P*<0.001) in the Em group compared with the control group, while HspA2 expression was decreased in Em+Cr and Cr groups compared with the Em group significantly in different cells of germinal epithelium including spermatogonia (*P*<0.001), primary spermatocytes (*P*<0.001), and spermatids (*P*<0.001), and there was decreased HspA2 expression in different cells of germinal epithelium including spermatogonia (*P*<0.001), primary spermatocytes (*P*<0.001), and spermatids (*P*<0.001) in the Cr group compared with the control group (Figures 9, 10A, 10B, and 10C). 

## Discussion

The present study was conducted to evaluate the protective effects of crocin on the male reproductive system against possibly harmful effects of electromagnetic field exposure. Today, the use of mobile phones has become a principal part of our lives (39). The rapid growth of using mobile phones has been accompanied by a parallel increase in the EMF density (40, 41). Some researchers have reported that long-term exposure to 900 MHz cell phones leads to a decrease in the weight of testis, the diameter of seminiferous tubules, the height of germinal epithelia, and the thickness of tunica albuginea (41-43). According to the results of the present study, testis weight index and testis volume decreased in the Em group and increased in Cr and Em+Cr groups, but these changes were not significant. In a similar study, it was found that long-term exposure to 900 MHZ EMF does not affect genital organ weights (testis, epididymis, prostate, and seminal vesicles) (43). Evaluation of this finding together with the reference sources suggests that EMF exposure does not cause a loss of weight in testis tissue.

In this study, seminiferous tubule diameter and germinal epithelium thickness were measured in order to reveal the effect of 2100 MHz EMF on these parameters. Seminiferous tubule diameter and germinal epithelium thickness were not changed significantly. This finding is supported by some studies, though not all. For example, other scientists reported no significant difference in seminiferous tubule diameter and seminiferous epithelium thickness between the EMF and control groups (44). A significant decrease was reported in seminiferous tubule diameter and germinal epithelium thickness in the EMF group (41). Perhaps the reason for the difference in our results and other studies is the short time exposure to electromagnetic field compared with the above studies. Because the frequency and duration of exposure to EMF are two important factors in the assessment of the impact of EMF (5).

Some research results showed that 2.45 GHz Wi-Fi significantly decreased the number of sperm and sperm motility (45). In the present study, it was also shown that exposure to EMF caused a decrease in the epididymal sperm count and an increase in the percentage of abnormal sperm. This studies confirmed our findings that EMF exposure reduces the number of sperm and increases the percentage of abnormal sperm. Of course in our study, with crocin administration, there was a significant increase in the epididymal sperm and a significant decrease in the percentage of abnormal sperm. Other studies have also confirmed our findings, for example, some scientists showed that crocin makes significant changes in reproductive indices and prevents the harmful effects caused by nicotine in the reproductive hormones; crocin could act as an antioxidant and improve the sperm quality probably due to crocin antioxidant activity (46). Increased sperm counts might possibly be induced by the anti-apoptotic effects of crocin (47). Crocin has shown anti-oxidant like activity *in vivo*, preventing oxidant-induced apoptosis, by preventing the formation of free radicals and lipid peroxidation (20).

The results of a study showed that exposure to electromagnetic radiation throughout embryogenesis may cause reductions in serum total testosterone, size, and weight of the male rat testis while causing a modest increase in apoptosis (31). Also, it was concluded that the serum levels of testosterone significantly decreased as a result of exposure to EMF. Serum LH and FSH levels also decreased as a result of exposure to EMF, but this reduction was not significant (42). Our study showed that EMF causes a reduction in serum level of LH, FSH, and testosterone, and with crocin administration, there was a significant increase in serum levels of LH, FSH, and testosterone. Other studies also confirmed our research results, for example, a study results revealed that crocin induced significant changes in the parameters of the reproductive system against nicotine consumption, as well as the prevention of nicotinic effects on reproductive hormones. These effects are probably due to the antioxidant effects of crocin (46).

 Other reports suggested that EMF cause impair spermatogenesis, germ cell degeneration, reduce daily total sperm production, and increase apoptosis in the germinal epithelium (48-51). Previous studies demonstrated that continuous exposure to EMF (duration- and dose-dependent) leads to apoptosis of spermatogonia cells (49, 50, 52). Other study results showed significantly fewer mature spermatogonia cells (spermatid and spermatozoa) in exposed mice than in controls (50). In our study, the number of spermatogonia, early spermatocyte, and spermatid was decreased. As shown in other studies, crocin has an anti-oxidant nature, that prevents the formation of free radicals and lipid peroxidation, hence, preventing oxidant-induced apoptosis (20). Studies have already been done showing that crocin reduces apoptosis in the germinal epithelium of seminiferous tubules in testicular tissue (33, 35). According to our results, which was in line with previous studies, exposure to EMF induces apoptosis in the spermatogenic cell line in the germinal epithelium in the seminiferous tubules (spermatogonia cells, primary spermatocytes, and spermatocytes). Crocin administration, as a therapeutic agent, can reduce the number of apoptotic cells in this cell line. As a result, the administration of crocin could prevent cells from apoptosis.

HspA2s protect cells against environmental hazards, such as heat, chemicals, and radiation. In response to heat shock and stress, these proteins are expressed and prevent damages, and there may be a relationship between the expression of these proteins and the level of apoptosis (53). In a study, Hsp70-1 and Hsp70-3 were detected in primary stages of spermatocytes, and this temporal expression was approximately associated with the apoptotic cells and it suggests that the expression of Hsp70-1/3 was related to the apoptosis observed in spermatocytes (54). Some members of the Hsp70 family are continuously expressed, and some are strongly induced by stress. Therefore, this protein is an important detector in a cell, tissue, or organ under a stress response (53, 55). HspA2, which is known as a member of Hsp70 family, has been identified to have a crucial role in male reproduction, including sperm-egg recognition, protein packaging and transportation, substitution of histones by protamines during spermiogenesis, inhibition of apoptosis, and removal of remaining cytoplasm during sperm maturation (13, 56, 57). In our study, consistent with previous studies, there was a significant increase in the expression of HspA2 in mice exposed to EMF. However, the administration of crocin as a treatment reduced the expression of HspA2 in the germinal epithelium of seminiferous tubules in the mice testis.

Regarding the findings of this and past studies, EMF exposure can affect by some testis histological features, serum biochemical parameters, and sperm parameters, whereas crocin can improve these features.

Cr: Crocin; Em: electromagnet field

Cr: Crocin; Em: electromagnet field

B. Comparison of the means of abnormal sperm percentage in different groups. The percentage of abnormal sperm was increased in the Em group compared with the control group (*P<0.001) and increased in Em+Cr and Cr groups compared with the Em group significantly (#*P*<0.05, δ*P*<0.05, respectively)

Cr: Crocin; Em: electromagnet field

Cr: Crocin; Em: electromagnet field

Cr: Crocin; Em: electromagnet field

Cr: Crocin; Em: electromagnet field

Cr: Crocin; Em: electromagnet field

## Conclusion

We found that exposure to electromagnetic fields 2 hr/ day during 16 days, cannot affect the weight and volume of the testis, the diameter of seminiferous tubules, and the thickness of the germinal epithelium. But it decreases the number of sperm and increases the abnormal sperm percent in mice epididymis. Also crocin had a protective effect and improved these parameters. EMF exposure reduces LH, FSH, and testosterone levels in the serum while crocin increases these parameters. In our study, it was proven that EMF exposure increases apoptotic spermatogonia, early spermatocyte, and spermatids, while crocin decreases these cells. At least EMF exposure leads to high-level expression of Hsp-A2 protein and crocin reduce Hsp-A2 expression. In summery EMF cause damage in the male reproductive system while crocin heals these damages.
